# Suicidality among adolescents and young adults in a Psychiatry Inpatient Unit: a two-year retrospective study in Umbria, central Italy

**DOI:** 10.1192/j.eurpsy.2023.727

**Published:** 2023-07-19

**Authors:** E. Valentini, A. Minuti, G. Menculini, F. De Giorgi, T. Sciarma, K. Amantini, P. Moretti, A. Tortorella

**Affiliations:** 1 Department of Psychiatry, University of Perugia; 2 Section of Psychiatry, Clinical Psychology and Rehabilitation, Santa Maria della Misericordia Hospital; 3 Psychiatric Inpatient Unit, Department of Mental Health, AUSL Umbria 1, PG, Italy

## Abstract

**Introduction:**

Suicide is the fourth leading cause of death among adolescents and young adults (AYA) (Czyz EK, King CA. *J Clin Child Adolesc Psychol.*2015;44(1):181-19), and psychiatric disorders are a major contributing factor (WHO Global Health Estimates 2000-2019). Studies focusing on suicidality in Italian inpatients samples are scant.

**Objectives:**

The present cross-sectional study aimed to define clinical variables associated with suicidality related phenomena in a sample of young inpatients. The main objectives were to assess the prevalence of suicidal ideation and deliberate self-harm in inpatients aged 16–24 years and to determine the relationship between suicidal behaviours and psychiatric disorders.

**Methods:**

This retrospective study was conducted in a naturalistic setting, at the Psychiatric Inpatient Unit, Perugia Hospital/Local Mental Health Unit 1, from January 2018 to December 2019. Sociodemographic information, clinical history, diagnostic and treatment features were collected. Descriptive and bivariate analyses were performed (p<0,05).

**Results:**

Among 120 patients (14,2% of the overall 850 hospitalizations in the index period) admitted for suicidality-related phenomena, 21 (17,5%) were AYA. Admission was due to deliberate self-harm in 85,7% (n=18) and to suicidal ideation in 14,3% (n=3) cases. Personality disorders (p=0.006), were significantly more prevalent among AYA, while mood disorders were more frequent among adults (p=0.0018) (Tab.1).

**Tab.1.** Differences in diagnostic features between AYA and adult population.

	AYA n (%)	Adults n (%)	χ^2^	P
PERSONALITY DISORDERS	10 (47.6)	17 (17,2)	7.547	0.006
Borderline personality disorder	7 (33.3)	9 (9.1)	6.838	0.009
MOOD DISORDERS	0 (0)	26 (26,3)	5.578	0.018

**Conclusions:**

Personality is under construction among youths, and affective symptoms may have unusual characteristics (Lack CW, Green AL. *J Pediatr Nurs.* 2009;24(1):13-25), as demonstrated by the fact that irritable rather than depressed mood is a core diagnostic mood symptom for adolescents (Rice F, et al. *J Affect Disord.* 2019;243:175-181). We hypothesized that symptoms of irritability, emotional dysregulation, and impulsivity could be linked to suicidality (Ghanem M, et al. *Arch Suicide Res.* 2013;17(3):262-274). Further investigations are needed for the characterization of AYA inpatients who experience suicidal thoughts or self-injurious behaviours, in order to redefine preventive tools and reduce suicide mortality rates.

**Disclosure of Interest:**

None Declared

